# A Study on Improvement of Solubility of Rofecoxib and its effect on Permeation of Drug from Topical Formulations

**DOI:** 10.4103/0250-474X.44595

**Published:** 2008

**Authors:** Madhur Kulkarni, Mangal Nagarsenker

**Affiliations:** Department of Pharmaceutics, Bombay College of Pharmacy, Kalina, Mumbai-400 098, India

**Keywords:** Rofecoxib, β-cyclodextrin, binary mixture, in vitro skin permeation

## Abstract

Rofecoxib, a practically insoluble cox-2 selective nonsteroidal antiinflammatory agent was subjected to improvement in solubility by preparing its binary mixtures with β cyclodextrin using various methods such as physical mixing, co-grinding, kneading with aqueous methanol and co-evaporation from methanol-water mixture. Characterization of the resulting binary mixtures by differential scanning calorimetry and X-ray diffraction studies indicated partial amorphization of the drug in its binary mixtures. *In vitro* dissolution studies exhibited remarkable increase in rate and extent of dissolution of the drug from its complexes with β -cyclodextrin. Pure rofecoxib as well as its co-ground binary mixture were formulated as aqueous gels for topical application. *In vitro* skin permeation of rofecoxib from formulation containing rofecoxib-cyclodextrin complex was significantly higher (p<0.05) at 1, 2, 12, 18 and 24 hr as compared to formulation containing pure rofecoxib. This could be attributed to better solubility of binary mixture in the aqueous gel vehicle leading to greater concentration gradient between the vehicle and skin and hence higher flux of the drug.

Rofecoxib (RXB), a cyclooxygenase -2 (cox-2) specific nonsteroidal antiinflammatory drug (NSAID) is indicated for the relief of signs and symptoms of osteoarthritis, rheumatoid arthritis and management of pain in adults^1^. Treatment of arthritis generally involves long term therapy with NSAIDs, chronic oral administration of which is associated with undesirable side effects especially gastrointestinal (GI) disorders. RXB in its therapeutic concentrations inhibits only cox-2 and by sparing cox-1 which is associated with maintaining the integrity of mucosal lining of GIT, shows lower incidences of GI side effects. However, chronic therapy with RXB has shown to result in clinical ulcers, gastrointestinal discomfort and bleeding and also in cardiovascular side effects such as hypertension, pedal edema and heartburn^2^. Hence, it would be beneficial to target RXB locally to the arthritic joints or inflamed area in order to achieve its optimum therapeutic effect and overcome the side effects encountered upon oral administration. Moreover, topical application of selective cyclooxygenase inhibitors has been shown to suppress ultra violet-B rays (UV-B)-mediated cutaneous inflammation^3^. Therefore, topical application of RXB may also aid in inhibiting light induced skin inflammation. Skin, irrespective of its protective barrier function can serve as a promising portal of entry of drugs for local as well as systemic action^4^. Flux of topically applied drug from a dermal delivery system into the skin depends on physicochemical properties of drug such as its molecular weight, partition coefficient and solubility as well as on the vehicle in which the drug is dissolved or suspended. Out of these factors, solubility is a very crucial factor in deciding the overall permeation of the drug.

Very poor aqueous solubility of RXB, could have an undesirable effect on its permeation across the skin. Hydrophilic cyclodextrins are known to improve the solubility of insoluble drugs by forming inclusion complexes^5^. Incorporation of the inclusion complexes in the aqueous based topical or transdermal formulations has many times exhibited improved skin permeation of poorly soluble drugs such as piroxicam^6^, flurbiprofen^7^, hydrocortisone^8^, miconazole^9^.

In aqueous topical formulations, cyclodextrins keep the lipophilic insoluble drug molecules in the form of saturated solution and deliver them to the skin surface in relatively larger concentration creating a greater concentration gradient across the skin and thus facilitating the partitioning of the drug into the skin^10^.

The aim of the present study was to evaluate the influence of β-cyclodextrin (BCD) on the solubility and *in vitro* dissolution characteristics of RXB as well as on the *in vitro* skin permeation of the drug when formulated into an aqueous gel.

## MATERIALS AND METHODS

RXB was a gift sample given by Lyka Labs ltd. (Mumbai, India). Carbomer 980(Carbopol 940) was a generous gift sample from B. F. Goodrich (Mumbai, India). BCD was purchased from S.A. Chemicals (Mumbai, India). Solvents for HPLC were purchased from S. D. Fine Chem (Mumbai, India). Other reagents and chemicals used were of analytical (AR) grade. After obtaining necessary approvals from Institutional Animal Ethics Committee, guinea pigs were procured from Hindustan Lever Ltd. (Mumbai, India) for the *in vitro* skin permeation studies.

### Solubility studies:

 Phase solubility study was carried out to investigate the effect of BCD on the solubility of RXB, using the method reported by Higuchi and Connors^11^. Plain distilled water containing no BCD and aqueous solutions of BCD (molecular weight - 1135) of different concentrations (2, 4, 6, 8, 10 and 15 mM) were added to excess amounts of RXB and shaken at 30° for 24 h. After equilibrium, the solutions were filtered through Whatmann® No.1 filter papers and diluted suitably to determine the concentration of RXB using Shimadzu 160A spectrophotometer. The graph of concentration of RXB was plotted against the concentration of β-cyclodextrin. Stability constant (Ks) for the complex was determined from the graph using following equation, Ks = slope/S_0_ (1-slope), where slope is obtained from the graph and S_0_ is equilibrium solubility of RXB in water.

### Preparation of binary mixtures (BMs):

BMs of RXB and BCD were prepared in the molar ratio of 1:1. Physical Mixture (PM) was prepared by geometric mixing of RXB and BCD without applying pressure. Co-ground BM (CG) was prepared by mixing and triturating RXB and BCD for 15 to 20 min. Co-evaporated BM (COEVAP) was prepared by dissolving RXB in methanol and BCD in water, following which both the solutions were mixed and the solvent mixture was evaporated by controlled heating at 45-50° with continuous stirring of solution until dry. Kneaded BM (KD) was prepared by geometric mixing of powders, RXB and BCD and then kneading with 1:1 mixture of methanol-water to obtain a mass with a pasty consistency, which was dried in a tray dryer at 45 to 50°. All the BMs were prepared in triplicate and were sieved through BSS 85# sieve (Sieve diameter 180 μ) and stored over anhydrous calcium chloride in dessicator.

### Characterization of BMs:

The Infra Red (IR)spectra, differential scanning calorimetry (DSC) thermograms and X-ray diffraction (XRD) patterns of RXB, BCD and the BMs were recorded. The IR spectra were recorded on a Jasco FT/IR 5300 spectrophotometer by potassium bromide (KBr) pellet method. For DSC studies, RXB, BCD and the BMs each weighing in the range of 3 to 5 mg were scanned at a rate of 10°/minute on a Shimadzu DT-40 Thermal Analyzer between 30° and 330° under inert atmosphere of nitrogen and the XRD patterns of RXB, BCD and their BMs were recorded using a Phillips X-ray Diffractometer (PW 1710) with a copper target, voltage 40 kV, current 30 mA at a scanning speed of 1° per minute.

### Assay and *in vitro* dissolution studies:

Each BM equivalent to 12.5 mg of RXB was weighed accurately and dissolved in 25 ml of methanol by sonication; 1 ml of this solution was diluted to 100 ml with 0.1 N HCl containing 0.5% polysorbate 80 (Tween 80). The drug content was calculated based on the absorbance of the solution at 262 nm. All the BMs were assayed in triplicate.

*In vitro* dissolution studies were performed in six replicates using USP Type 2 apparatus in 900 ml of 0.1N HCl containing 0.5% Tween 80 as medium maintained at 37±0.5° using the speed of 50±2 rpm. RXB, 12.5 mg or equivalent quantity of BM was weighed accurately and dispersed in the dissolution medium. Aliquots were withdrawn at regular time intervals, filtered using Whatmann® No.1 filter papers, suitably diluted and read spectrophotometrically at 262 nm.

### Preparation of gel formulations:

Table 1 illustrates the formulae used for the preparation of gel formulations of RXB. The formulations were prepared using 0.5% w/w of RXB or equivalent amount of CG i.e. the binary mixture prepared by co-grinding method. Weighed quantity of Carbopol 940 (carbomer 980), was dispersed in measured quantity of hot water (50-60°) under continuous stirring. Sodium benzoate was dissolved in the above dispersion and the carbomer was neutralized by addition of suitable quantity of triethanolamine solution under stirring to get a transparent gel. The pH of the gel base was maintained between 5.3 and 5.4. Weighed quantity of drug or BM was triturated with distilled water to obtain homogenous slurry in case of formulations F1 and F2 respectively. Previously prepared gel base was added to the slurry of RXB or BM and mixed using an overhead stirrer (Remi, Mumbai) for sufficient time to get a homogeneous white colored gel formulation. The prepared formulations were filled in lacquered aluminum collapsible tubes and stored in cool place.

**TABLE 1:** COMPOSITION OF TOPICAL GEL FORMULATIONS OF RXB AND BM

Ingredients	F1 g	F2 g
RXB	0.5	-
BM	-	2.3
Carbopol 940	0.7	0.7
Sodium benzoate	0.2	0.2
Distilled water	q.s.100	q.s.100
Triethanolamine	q.s. to achieve	q.s. to achieve
solution (50% v/v)	pH 5.3 to 5.4	pH 5.3 to 5.4

### Microscopic studies of gels:

Small quantities of formulations F1 and F2 were spread in the form of smears on the glass slides, observed under microscope and their photomicrographs were taken using Panasonic-GTKR222E camera attached to Hund Wetznar H-500 microscope.

### Assay of the formulations:

The formulations were assayed for RXB content in triplicate by validated reverse phase HPLC technique. The accurately weighed quantity of formulation was dissolved in suitable quantity of n, n dimethylformamide, diluted suitably with mobile phase and analyzed using an isocratic system involving JASCO PU- 2080 Plus intelligent pump, Jasco UV-2075 Plus UV/Vis detector set at 262 nm, Star 800 Module interface and Borwin software (version 1.5). Chromatograms were obtained by injecting samples on to the system equipped with 20μl loop and C_18_ HiQsil, 5μ column (250×4.6 mm) stabilized by the mobile phase consisting of acetonitrile: water (60:40) at the flow rate of 1ml/min.

### *In vitro* skin permeation studies:

Skin permeation studies were carried out using the Erweka apparatus (Munich, Germany) and Keshary Chien diffusion cells. For each formulation, the skin permeation was studied in 4 replicates. Full thickness abdominal skin was excised from adult albino guinea pigs weighing in the range of 300 to 400 g. The visceral side of the freshly excised skin was cleaned free of any adhering subcutaneous tissue. The hair on the epidermal surface of the skin was cut with the help of a pair of scissors, as close to skin as possible without damaging the skin. The diffusion cells had receptor compartment volume of 8 ml and diffusion area of 5 cm^2^. Phosphate buffer saline pH 7.4 was filled in receptor compartment and stirred continuously with the help of a magnetic stirrer. The medium was maintained at 37° with the help of a water jacket of the receptor compartment receiving water at the temperature of 37° from circulating water bath. The skin of suitable size was cut and placed properly between the donor and the receptor compartment with the dermal side in contact with the receptor medium. On the epidermal side of the skin, weighed amount of the formulation was spread evenly and both the donor and receptor compartments were clamped together. The donor compartment was covered with the aluminum foil tightly so as to avoid evaporation of water from the gel formulation during the study. The receptor medium was withdrawn completely and replaced with fresh medium at 1, 2, 4, 6, 8, 12, 18 and at the end of study duration i.e. at 24 h.

Samples were diluted suitably and analyzed spectrophotometrically for the content of RXB at 255 nm. The method of analysis was validated previously for specificity and linearity. Cumulative amount of RXB permeated through skin was plotted as a function of time. Permeation profiles of both the formulations were compared applying unpaired, two tailed Student’s t test at 5% level of significance.

## RESULTS AND DISCUSSION

**Fig. 1:**
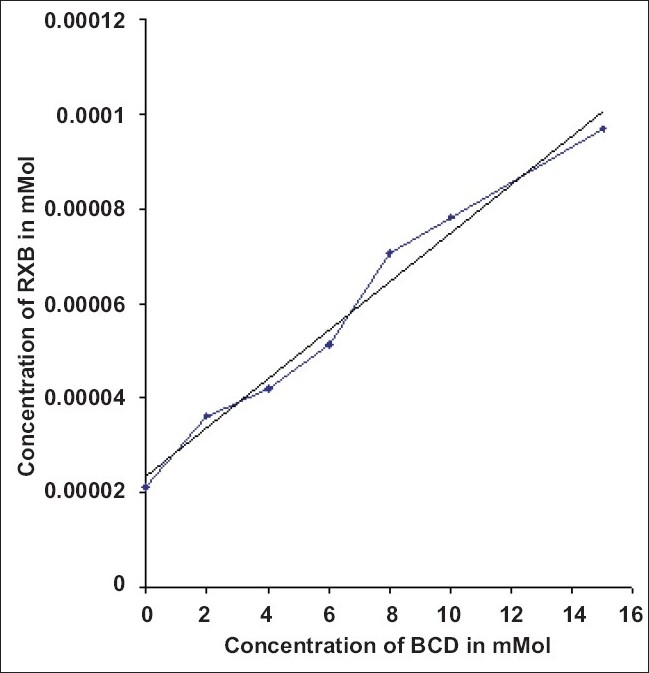
Phase solubility study of RXB in solutions of BCD of various concentrations; The equation obtained was 5E-06x + 2E-05 with a r^2^ value of 0.9798. RXB is rofecoxib and BCD is β-cyclodextrin

Present work involved determining the influence of BCD on the solubility of RXB. The phase solubility diagram (fig. 1) could be classified as Type A_L_ according to Higuchi and Connors. As the slope of the line was less than unity, it was assumed that the increase in solubility observed was due to the formation of a 1:1 complex. The apparent stability constant K_1: 1_ was found to be238 M^-1^ indicating adequate stability of the complex.

**Fig. 2:**
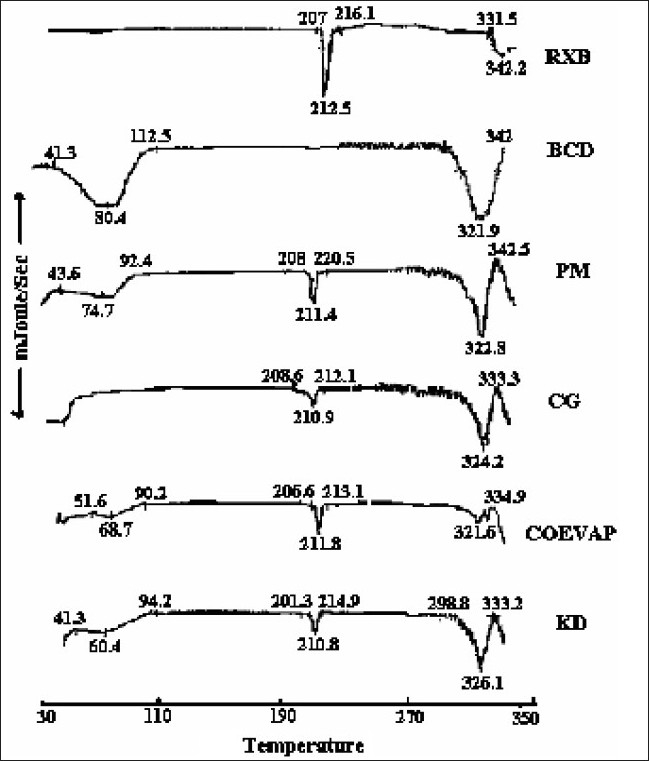
DSC scans of RXB, BCD and their BMs

**Fig. 3:**
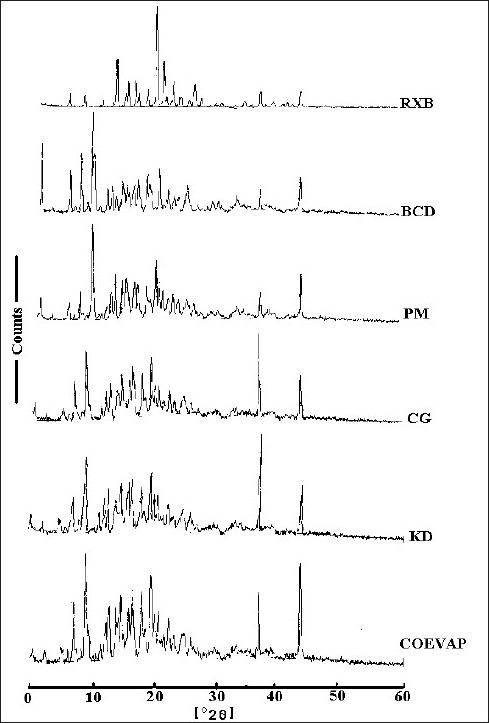
XRD scans of RXB, BCD and their BMs

RXB, BCD and their BMs, were subjected to IR spectroscopy to determine any possible interaction of the drug with the carrier. There were no significant changes in the IR spectra of the BMs when compared with that of the pure RXB thus indicating minimal chemical interaction between the drug and BCD as a result of preparation of BMs. DSC scan of RXB showed a sharp melting endotherm at 212.5° (fig. 2). Scan of BCD showed a broad endotherm in the range of 40 to 112° indicating loss of water of crystallization during heating and a melting endotherm in the range of 290 to 330°. A shallow endotherm was seen in the range of 205 to 217° in all the BMs indicating partial amorphization of the drug due to drug BCD interaction. Endotherm indicating loss of water as observed in the scan of BCD was either very shallow or even absent in case of BMs where as endotherm indicating melting of BCD was present. During formation of inclusion complex between RXB and BCD; one of the non-polar groups of the drug having more affinity for the hydrophobic cavity of BCD molecule could have replaced the otherwise entrapped water from the cavity which resulted in absence of the endotherm for evaporation of water in the thermograms of the BMs. XRD scan of RXB showed intense peaks of crystallinity. Scans of PM, COEVAP, CG and KD showed decreasingly lesser number of peaks with lower intensity indicating partial amorphization of the drug in its BMs (fig. 3). X- Ray scan of KD showed greater amorphization of the drug. The assay values of all the BMs were in the close range of 98 to 102% with RSD less than 2% thus indicating uniformity of drug distribution in BCD carrier.

**Fig. 4:**
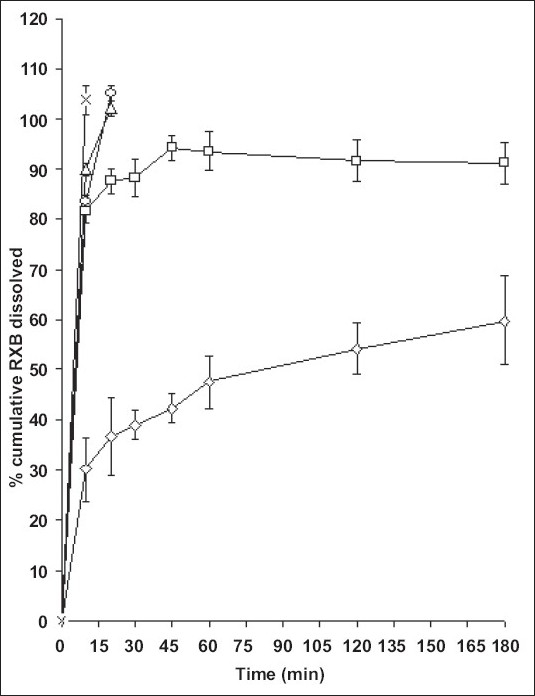
*In vitro* dissolution studies of RXB and its BMs Each point is mean±sd of six determinations. RXB (–◊–), PM (–□–), CG (–△–), COEVAP (–○–) and KD (–×–)

Minimum therapeutic dose of RXB for oral administration is 12.5 mg hence *in vitro* dissolution studies were carried out for the same quantity of the drug. RXB powder, when studied for *in vitro* dissolution in 0.1N HCl containing 0.5% Tween 80 exhibited only 60% dissolution at the end of 3 hours with a large variation in amount dissolved at each time interval (fig. 4). PM although just a blend of RXB and BCD exhibited 93% release at the end of 3 h which could be due to *in situ* interaction between drug and the cyclodextrin during dissolution studies leading to formation of a complex with improved solubility. CG showed complete release within 20 min indicating good association of RXB and BCD attainable without involving any solvent but just by grinding of the two powders. KD exhibited fastest release with complete drug dissolution in 10 min. Thus, preparation of BMs significantly improved rate as well as the extent of *in vitro* dissolution of the drug owing to the factors such as complexation, partial amorphization of the drug and its improved wettability in presence of BCD.

**Fig. 5:**
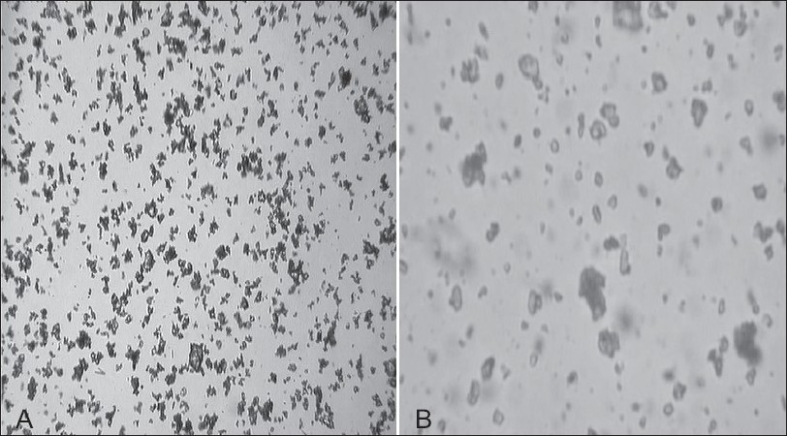
Photomicrographs of gel formulations Photomicrographs of A. Gel F1 containing RXB (rofecoxib) and B. Gel F2 containing BM (binary mixtures)

Aqueous based gel formulations are well-accepted dosage forms for the topical application owing to their elegant appearance, cooling effect, good spreadability, non-tackyness and ease of removal. Hence, gel was chosen as a vehicle for the development of topical formulation of RXB. To evaluate the influence of improved solubility of the drug on its permeation characteristics, CG equivalent to 0.5% of RXB was formulated in to the same gel base. pH of the formulation was maintained between 5.3 to 5.4 which is close to the physiological pH of the skin and it would not affect the stability of the drug. Sodium benzoate, a preservative effective in acidic pH was added. The formulations were observed under microscope to determine the distribution and morphological characteristics of the drug in the gel vehicle. In case of system F1, fairly uniform distribution of small agglomerates of drug particles in the gel base indicated homogeneous mixing of the drug in the base. Formulation F2 containing BM showed almost spherical shaped fine drug particles with smooth edges indicating altered morphology of the drug in its BM with cyclodextrin (fig. 5). The gel formulations were assayed by reverse phase HPLC method that was previously validated for specificity, linearity, accuracy, precision, intra and inter day variation. The drug content of both the formulations was within the range of 95 to 105%.

**Fig. 6:**
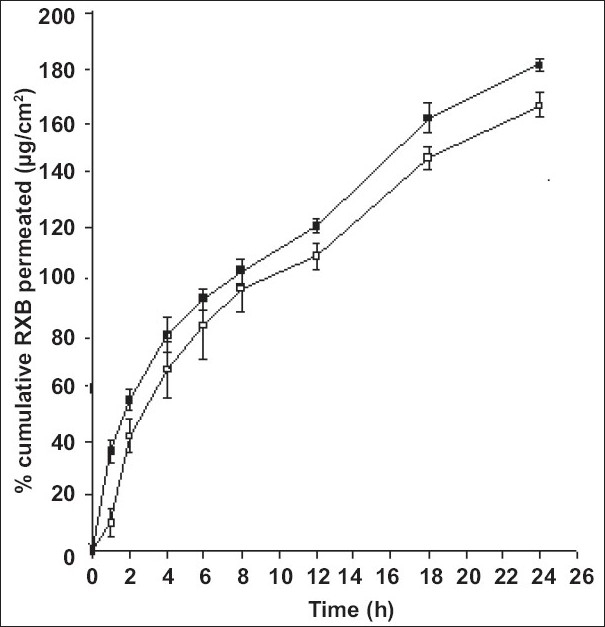
*In vitro* skin permeation profiles of RXB from gel formulations F1 and F2 through guinea pig skin Each point is mean±sd of six determinations. Gel F1 containing rofecoxib (RXB) (—□—), Gel F2 containing binary mixtures (BM) (—■—)

Abdominal skin of guinea pigs is widely used for the permeation studies of the topical formulations^12^,^13^. Hence, permeation experiments of RXB from the formulations were conducted using guinea pig skin. Comparative permeation profiles of both the formulations are presented in fig. 6. Permeation of RXB from system F2 was found to be overall better than from the formulation F1 and significantly better (p<0.05) at 1, 2, 12, 18 and 24 h intervals which could be attributed to improved solubility of RXB in its BM resulting in building better concentration gradient and hence higher diffusion between vehicle and the skin surface leading to greater permeation of the drug. Average steady state flux values determined from the linear portions of the graph for formulation F1 and F2 were 5.457 and 6.0512 μg cm^-2^ h^-1^, respectively.

In conclusion, BMs of RXB and BCD prepared by various methods exhibited improvement in apparent aqueous solubility and *in vitro* dissolution profile of the drug owing to partial amorphization of drug as revealed by DSC and XRD studies. Incorporation of CG in to the gel formulation resulted in better *in vitro* skin permeation of drug as compared to the system containing drug alone thus confirming the beneficial effect of BCD on permeation of the drug.
